# Docetaxel induced-JNK2/PHD1 signaling pathway increases degradation of HIF-1α and causes cancer cell death under hypoxia

**DOI:** 10.1038/srep27382

**Published:** 2016-06-06

**Authors:** Eun-Taex Oh, Chan Woo Kim, Soo Jung Kim, Jae-Seon Lee, Soon-Sun Hong, Heon Joo Park

**Affiliations:** 1 Department of Biomedical Sciences, College of Medicine, Inha University, Incheon 22212, Republic of Korea; 2 Hypoxia-related Disease Research Center, College of Medicine, Inha University, Incheon 22212, Republic of Korea; 3 Department of Microbiology, College of Medicine, Inha University, Incheon 22212, Republic of Korea; 4 Department of Molecular Medicine, College of Medicine, Inha University, Incheon 22212, Republic of Korea

## Abstract

HIF-1 (hypoxia-inducible factor-1) regulates the expression of more than 70 genes involved in angiogenesis, tumor growth, metastasis, chemoresistance, and radioresistance. Thus, there is growing interest in using HIF-1 inhibitors as anticancer drugs. Docetaxel, a Food and Drug Administration-approved anticancer drug, is reported to enhance HIF-1α degradation. Here, we investigated the molecular mechanism underlying docetaxel-induced HIF-1α degradation and cancer cell death under hypoxic conditions. Docetaxel pretreatment enhanced the polyubiquitination and proteasome-mediated degradation of HIF-1α, and increased cancer cell death under hypoxic conditions. Docetaxel also activated the prolyl hydroxylase, PHD1, in hypoxia, and pharmacological inhibition or siRNA-mediated knockdown of PHD1 prevented docetaxel-induced HIF-1α degradation and cancer cell death. Additionally, siRNA-mediated JNK2 knockdown blocked docetaxel-induced HIF-1α degradation and cancer cell death by inhibiting PHD1 activation. A luciferase reporter assay revealed that inhibition of the JNK2/PHD1 signaling pathway significantly increased the transcriptional activity of HIF-1 in docetaxel-treated cancer cells under hypoxia. Consistent with these results, docetaxel-treated JNK2-knockdown tumors grew much faster than control tumors through inhibition of docetaxel-induced PHD1 activation and degradation of HIF-1α. Our results collectively show that, under hypoxic conditions, docetaxel induces apoptotic cell death through JNK2/PHD1 signaling-mediated HIF-1α degradation.

Docetaxel is a semi-synthetic taxoid derived from the European yew (*Taxus baccata*), and the first promising paclitaxel analog for cancer treatment^1^. It has been used to treat gastric, breast, ovarian and non-small cell lung cancers^1^. Paclitaxel and docetaxel inhibit G2/M progression through destabilization of the microtubule network, which is required for spindle formation during mitosis^2^,^3^,^4^; as such, these agents may be useful for radiosensitization of cancer cells^4^,^5^,^6^,^7^. However, whereas docetaxel is cytotoxic toward hypoxic cancer cells, paclitaxel is relatively ineffective against these cells^4^. Previous work has revealed that docetaxel is cytotoxic toward prostate cancer cells under hypoxic conditions because it inhibits accumulation of hypoxia-inducible factor (HIF)-1α^8^. Additionally, it has been reported that docetaxel regulates HIF-1α protein expression, but not mRNA^9^. However, how it regulates HIF-1α protein expression and cancer cell death under hypoxic conditions is unclear.

Hypoxia is a common characteristic of the tumor microenvironment that triggers the HIF signaling pathway in cancer cells, thereby increasing angiogenesis, tumor growth, metastasis, chemoresistance, and radioresistance^9^,^10^,^11^,^12^,^13^,^14^. Furthermore, overexpression of HIFs is reported to occur in ~70% of human tumors relative to adjacent normal tissue, making HIFs attractive targets for anticancer therapies^15^,^16^. HIFs are heterodimeric transcription factors that consist of one of three oxygen-regulated α-subunits, HIF-1α, HIF-2α and HIF-3α, and a constitutively expressed hydrocarbon receptor/nuclear translocator β subunit, HIF-1β^17^,^18^,^19^. Formation of a HIF-1α/HIF-1β heterodimer is required for HIF-1 function, as HIF-1α serves as the major regulatory subunit responsible for its transcriptional function^20^. Under normoxic conditions, HIF-1α is rapidly degraded (half-life <5 min) by the proteasome^21^. This occurs through HIF-1α hydroxylation on proline residues 402 and 564 by specific prolyl hydroxylases (PHD1–3) in the presence of oxygen, α-ketoglutarate, and iron^20^,^22^,^23^,^24^,^25^. The von Hippel-Lindau (pVHL) protein, which functions as an E3 ligase, binds to hydroxylated HIF-1α and recruits an E3 ubiquitin ligase complex that includes elongin-B, elongin-C and cullin2, thereby promoting ubiquitination and 26S proteasome-mediated degradation of HIF-1α^13^,^14^,^19^. In hypoxic conditions, HIF-1α is not hydroxylated, thereby preventing its interaction with pVHL and subsequent ubiquitination and proteasomal degradation^8^. Following hypoxic stabilization, HIF-1α is translocated to the nucleus with HIF-1β and binds to hypoxia-response elements (HREs), thereby increasing the transcription of approximately 70 genes involved in cell adaptation and survival under hypoxic conditions^8^,^10^,^11^,^26^. Recently, it was reported that HIF-1α activity is controlled by O_2_/PHD/VHL-independent mechanisms through the regulation of various proteins, including receptor of activated protein kinase C 1 (RACK1), heat-shock protein 70 (HSP70), carboxyl terminus of HSP70-interacting protein (CHIP), Cullin-5, c-Jun N-terminal kinase 1 (JNK1), B-cell lymphoma 2 (Bcl2), Runt-related transcription factor 2 (Runx2), and factor inhibiting HIF-1 (FIH-1)^14^,^19^,^20^,^27^,^28^,^29^.

The purpose of the present study was to elucidate the mechanism by which docetaxel regulates HIF-1α expression and induces cancer cell death under hypoxic conditions. We show that docetaxel dose-dependently inhibits HIF-1α accumulation through a mechanism that involves increased phosphorylation/activation of JNK2, which phosphorylates and activates PHD1. Activated PHD1, in turn, decreases HIF-1α stability in hypoxic cancer cells by promoting its ubiquitin-proteasome-mediated degradation, thereby inducing cancer cell death. Collectively, these findings reveal a novel mechanism that mediates the regulation of HIF-1α and anticancer effects of docetaxel in hypoxic cancer cells.

## Results

### Effects of docetaxel on HIF-1α expression and transcriptional activation in cancer cells under hypoxia

**Figure 1:**
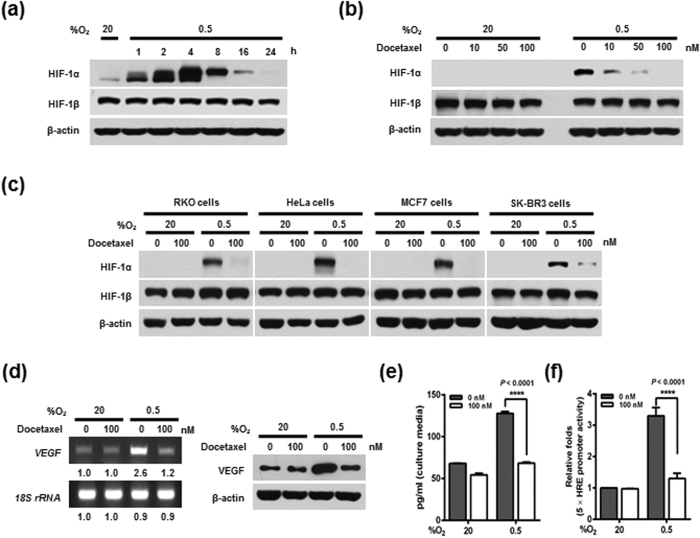
Effects of docetaxel on HIF-1α expression and transcriptional activation in cancer cells under hypoxia. (**a**) MDA-MB-231 cells were exposed to 0.5% O_2_ for 24 h and harvested at the indicated times. Whole-cell lysates were analyzed by immunoblotting for the indicated proteins. (**b**) MDA-MB-231 cells were treated with different concentrations of docetaxel (0–100 nM) for 16 h and exposed to 20% or 0.5% O_2_. After a 4-h incubation, cells were harvested, and cell lysates were analyzed by immunoblotting for the indicated proteins. (**c**) RKO, HeLa, MCF7, and SK-BR3 cells were incubated with or without 100 nM docetaxel for 16 h and exposed to 20% or 0.5% O_2_. After a 4-h incubation, cells were harvested, and whole-cell lysates were analyzed by immunoblotting for the indicated proteins. (**d**) MDA-MB-231 cells were incubated with or without 100 nM docetaxel for 16 h, exposed to 20% or 0.5% O_2_ for 24 h, and then harvested. RT-PCR (left panel) was used to amplify *VEGF* mRNA and *18S rRNA*, and immunoblot analysis (right panel) was used to detect VEGF and β-actin proteins. Band intensities of RT-PCR products from cells cultured under hypoxic conditions relative to those from cells in normoxic conditions were quantified using Image J (NIH). (**e**) MDA-MB-231 cells were incubated with or without 100 nM docetaxel for 16 h and exposed to 20% or 0.5% O_2_. After a 24-h incubation, conditioned media were harvested. Secreted VEGF in conditioned media was analyzed by ELISA. Data are presented as means ± SD (****P < 0.0001; ANOVA). (**f**) MDA-MB-231 cells co-transfected with p5 × HRE-*luc* and pCMV-β-galactosidase were cultured for 16 h, then incubated with or without 100 nM docetaxel for 16 h, and exposed to 20% or 0.5% O_2_ for 4 h. Luciferase activity was normalized to that of β-galactosidase. Data are presented as means ± SD (****P < 0.0001; ANOVA).

HIF-1α is stabilized under hypoxic conditions, whereas HIF1β, its binding partner in a heterodimeric complex, is constitutively expressed in normoxic and hypoxic cancer cells^17^,^18^,^19^. Although HIF-1α is stabilized under acute hypoxia, prolonged hypoxia leads to decay of HIF-1α^19^. To determine the effects of hypoxia on HIF expression in cancer cells, we exposed MDA-MB-231 cells to normoxia (20% O_2_) or hypoxia (0.5% O_2_) for 24 h, and assessed HIF-1α and HIF-1β expression by immunoblot analysis. Consistent with previous reports, HIF-1α expression was markedly increased in cells exposed to 0.5% O_2_, exhibiting a peak increase at 4 h followed by a gradual decline in expression, whereas HIF-1β levels were unchanged (Fig. 1a). To investigate the effects of docetaxel on HIF expression in cancer cells, we treated MDA-MB-231 cells with docetaxel (0–100 nM) for 16 h, and further exposed cells to 20% or 0.5% O_2_ for 4 h. Docetaxel caused a dose-dependent decrease in HIF-1α expression under hypoxic conditions, but did not alter HIF-1β levels (Fig. 1b). Similar results were observed in RKO, HeLa, MCF7, and SK-BR3 cancer cell lines (Fig. 1c). We next evaluated the effects of docetaxel on the transcriptional activity of HIF-1α, which regulates numerous hypoxia-adaptive genes that contain HREs in their promoters^19^. To this end, we treated MDA-MB-231 cells with 100 nM docetaxel, and investigated the HIF-1α target gene, vascular endothelial growth factor (*VEGF*), using reverse transcription-polymerase chain reaction (RT-PCR), immunoblot analysis, and enzyme-linked immunosorbent assays (ELISAs). Under hypoxic conditions, docetaxel treatment decreased the mRNA and protein levels of VEGF in cells (Fig. 1d,e). To confirm these data, we transfected MDA-MB-231 cells with a reporter plasmid (p5 × HRE-*luc*) and pCMV-β-galactosidase (transfection control), treated them with docetaxel, and then exposed them to 20% or 0.5% O_2_ for 4 h. We found that docetaxel treatment reduced luciferase activity by ~50% under hypoxic conditions (Fig. 1f). Taken together, these observations indicate that treatment of hypoxic cells with docetaxel decreases HIF-1α expression, thereby decreasing HIF-1α–mediated transcription and expression of downstream target genes.

### Docetaxel decreases HIF-1α protein stability in cancer cells under hypoxia

**Figure 2:**
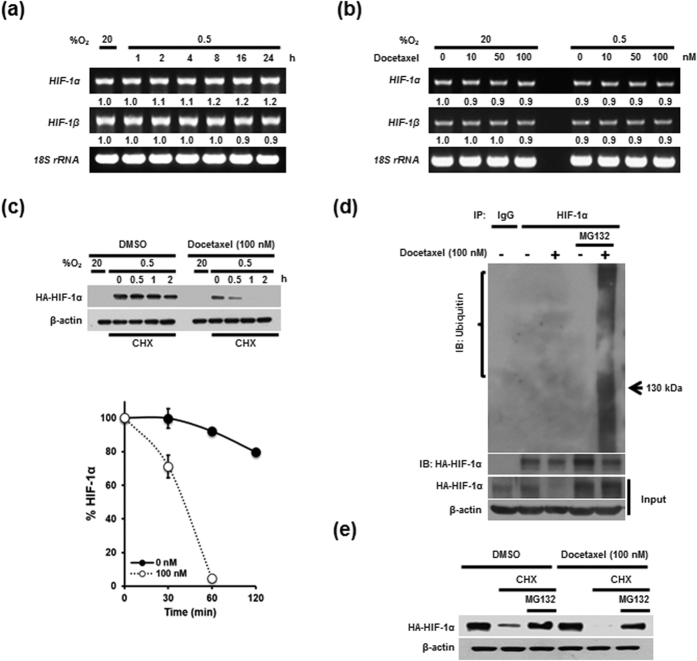
Docetaxel decreases HIF-1α protein stability in cancer cells under hypoxia. (**a**) MDA-MB-231 cells were exposed to 0.5% O_2_ for 24 h and harvested at the indicated times. RT-PCR (left panel) was used to amplify *HIF-1α* and *HIF-1β* mRNA and *18S rRNA*. Band intensities of RT-PCR products from cells cultured under hypoxic conditions relative to those from cells in normoxic conditions were quantified using Image J. (**b**) MDA-MB-231 cells were treated with different concentrations of docetaxel (0–100 nM) for 16 h and exposed to 20% or 0.5% O_2_. After a 4-h incubation, cells were harvested. RT-PCR (left panel) was used to amplify *HIF-1α* and *HIF-1β* mRNA and *18S rRNA*. Band intensities of RT-PCR products from cells cultured under hypoxic conditions relative to those from cells in normoxic conditions were quantified using Image J. (**c**) MDA-MB-231 cells were transfected with pHA-pHIF-1α and incubated with or without 100 nM docetaxel for 16 h. Cells were exposed to 0.5% O_2_ for 4 h, incubated with 10 μg/ml CHX for 2 h, and then harvested at the indicated times. HIF-1α protein levels were examined by immunoblot analysis; β-actin was used as an internal control. HIF-1α protein levels were quantified using Image J, and band intensities were normalized to those of β-actin (band intensity at t_0_ was defined as 100%). (**d**) MDA-MB-231 cells were transfected with pHA-HIF-1α, and incubated with or without 100 nM docetaxel for 16 h. Cells were then exposed to 0.5% O_2_ for 4 h, and incubated for 1 h in the presence or absence of MG132. Whole-cell extracts were immunoprecipitated with an anti-HA antibody, and ubiquitinated HIF-1α was detected with an anti-ubiquitin antibody. (**e**) MDA-MB-231 cells were transfected with pHA-HIF-1α, then incubated with or without 100 nM docetaxel for 16 h. Cells were exposed to 0.5% O_2_ for 4 h, incubated with or without CHX and MG132 for 1 h, and harvested. Collected cells were analyzed by immunoblotting for the indicated proteins.

Previous studies have presented conflicting results regarding the effects of hypoxia on *HIF-1*α transcription, with one showing that exposure of human Hep3B cells to hypoxia caused a time-dependent increase in *HIF-1*α mRNA levels^21^ and another reporting that *HIF-1*α mRNA levels were unchanged by hypoxia^30^. Consistent with this latter report, we found that hypoxia had no effect on *HIF-1*α mRNA levels (Fig. 2a). To determine how docetaxel regulates HIF-1α expression, we quantified *HIF-1*α mRNA levels in MDA-MB-231 cells following treatment with different concentrations of docetaxel. As shown in Fig. 2b, we found that *HIF-1*α mRNA levels were unaffected by docetaxel treatment under both normoxic and hypoxic conditions. We therefore investigated whether docetaxel affects the accumulation and stability of HIF-1α protein. We first transfected MDA-MB-231 cells with a vector expressing HA-tagged HIF-1α protein (pHA-HIF-1α), treated them with docetaxel for 16 h, and exposed them to 0.5% O_2_ for 4 h to obtain maximum HIF-1α expression. The cells were then incubated with 10 μg/ml cycloheximide (CHX), which blocks *de novo* protein synthesis, and the decay in HIF-1α protein over time was measured by immunoblotting. HIF-1α was dramatically degraded within 1 h in the presence of docetaxel, whereas HIF-1α levels remained little changed in controls after 2 h (Fig. 2c). A previous report found that HIF-1α degradation is regulated by the ubiquitin-proteasome system^19^. To examine whether docetaxel increases ubiquitination and proteasome-mediated degradation of HIF-1α under hypoxic conditions, we transfected MDA-MB-231 cells with pHA-HIF-1α and treated them with docetaxel. After 16 h, the cells were exposed to 0.5% O_2_ and incubated with or without the proteasome inhibitor MG132. Cell extracts were immunoprecipitated with an anti-HA antibody, and levels of ubiquitinated HIF-1α in immunoprecipitates were assessed by immunoblotting using an anti-ubiquitin antibody. As shown in Fig. 2d, docetaxel increased HIF-1α ubiquitination in MG132-treated cell lines. To investigate whether docetaxel increases HIF-1α degradation via the ubiquitin-mediated proteasomal pathway under hypoxic conditions, we transfected MDA-MB-231 cells with pHA-HIF-1α and treated them with docetaxel. After 16 h, cells were exposed to 0.5% O_2_ and treated with CHX and/or MG132. As shown in Fig. 2e, MG132 treatment inhibited docetaxel-induced degradation of HIF-1α under hypoxic conditions. Collectively, these results demonstrate that docetaxel increases HIF-1α degradation via the ubiquitin-mediated proteasome pathway in hypoxic cells.

### Phosphorylation of PHD1 is required for docetaxel-induced HIF-1α degradation in cancer cells under hypoxia

**Figure 3:**
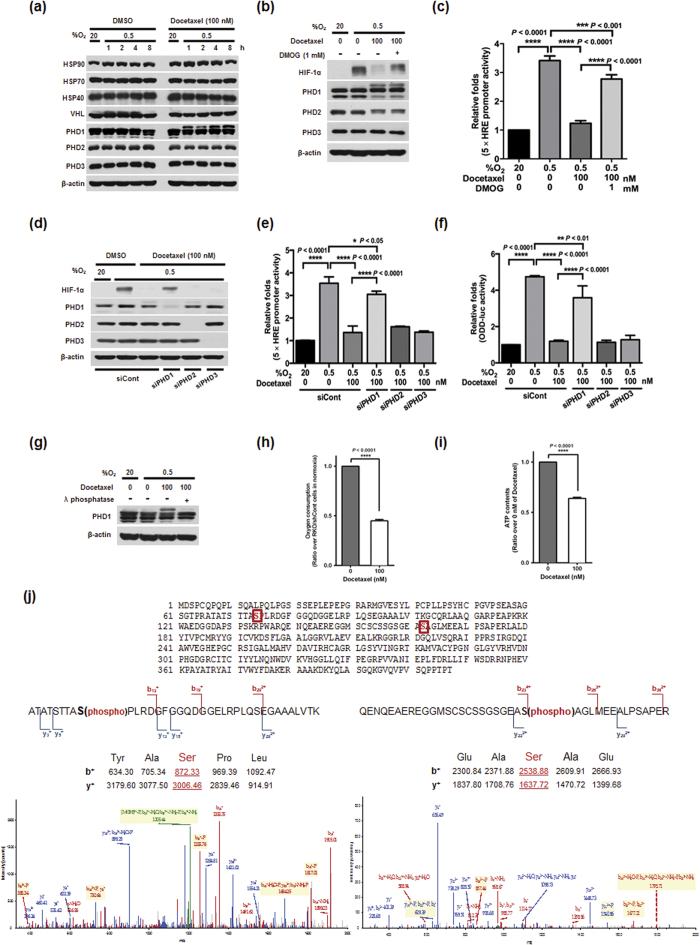
Phosphorylation of PHD1 is required for docetaxel-induced HIF-1α degradation in cancer cells under hypoxia. (**a**) Effect of docetaxel on the expression of HSP90, HSP70, HSP40, VHL, PHD1, PHD2, PHD3 and β-actin in MDA-MB-231 cells under hypoxia. (**b**) Effect of docetaxel on the expression of PHD1, PHD2, PHD3 and β-actin in MDA-MB-231 cells treated with or without DMOG (1 mM) under hypoxia. (**c**) Effect of docetaxel on the transcriptional activity of HIF-1α in MDA-MB-231 cells treated with or without DMOG under hypoxia. Luciferase activity was normalized to that of β-galactosidase. Data are presented as means ± SD (***P < 0.001, ****P < 0.0001; ANOVA). (**d**) Effect of docetaxel on the expression of HIF-1α, PHD1, PHD2, PHD3 and β-actin in MDA-MB-231 cells transfected with siRNA targeting PHD1, PHD2 or PHD3 under hypoxia. (**e**) Effect of docetaxel on the transcriptional activity of HIF-1α in siRNA for PHD1, PHD2 or PHD3-transfected MDA-MB-231 cells under normoxic and hypoxic conditions. Luciferase activity was normalized to that of β-galactosidase. Data are presented as means ± SD (*P < 0.05, ****P < 0.0001; ANOVA). (**f**) Effect of docetaxel on the hydroxylation activity of HIF-1α in siRNA for PHD1, PHD2 or PHD3-transfected MDA-MB-231 cells under normoxic and hypoxic conditions. Luciferase activity was normalized to that of β-galactosidase. Data are presented as means ± SD (**P < 0.01, ****P < 0.0001; ANOVA). (**g**) Effect of docetaxel on the phosphorylation of PHD1. (**h,i**) Effect of docetaxel on O_2_ consumption (**h**) and ATP generation (**i**) in MDA-MB-231 cells under hypoxia. Values are means ± SD from three experiments (****P < 0.0001; ANOVA). (**j**) LC-LTQ-Orbitrap spectrometer analysis of in-gel-digested PHD1 allowed the identification of PHD1 (UniProt Q96KS0) with 90% sequence coverage (searched against the UniProt human proteome database). Phosphorylated serine resides are denoted in red. ES-FTMS product ion spectra of the triply charged ion at m/z value 1237.60767 and the quadruply charged ion at m/z value 1002.90735. The triply charged ion and the quadruply charged ion correspond to the tryptic peptides ATATSTTAS(phospho)PLRDGFGGQDGGELRPLQSEGAAALVTK (lower left panel) and QENQEAEREGGMSCSCSSGSGEAS(phospho)AGLMEEALPSAPER (lower right panel), respectively.

Previous reports have shown that expression of the heat-shock protein HSP90 increases HIF-1α stability, whereas HSP40, HSP70, PHD1, PHD2, PHD3, and VHL decrease it^13^,^14^. Therefore, we investigated whether docetaxel affects the expression of these proteins during hypoxia. As shown in Fig. 3a, the levels of these proteins were not altered, regardless of oxygenation status. However, we found that docetaxel treatment increased a slower-migrating PHD1 band under hypoxic conditions, but did not affect PHD2 or PHD3 (Fig. 3a). We hypothesized that the slower-migrating PHD1 band was the activated form of PHD1. To test this, we determined whether PHD1 participated in docetaxel-induced HIF-1α degradation in hypoxic cancer cells. To this end, we treated MDA-MB-231 cells with dimethyloxalylglycine (DMOG), an inhibitor of PHDs, in the presence or absence of 100 nM docetaxel, and exposed these cells to 0.5% O_2_ for 4 h. As shown in Fig. 3b, under hypoxic conditions, DMOG prevented the docetaxel-induced decrease in HIF1α expression. To further confirm this, we transfected MDA-MB-231 cells with p5 × HRE-*luc* and pCMV-β-galactosidase, treated them with docetaxel, and exposed them to 20% or 0.5% O_2_ for 4 h. Under hypoxic conditions, DMOG treatment increased luciferase activity in the presence of 100 nM docetaxel (Fig. 3c). To define the potential contribution of PHDs to the regulation of HIF-1α in docetaxel-treated cells under hypoxic conditions, we transfected MDA-MB-231 cells with small interfering RNAs (siRNAs) targeting PHD1 (siPHD1), PHD2 (siPHD2) or PHD3 (siPHD3). We then exposed these cells to 0.5% O_2_ for 4 h and assessed HIF-1α expression/hydroxylation by immunoblotting and p*ODD-luc* assay. siPHD1 blocked the docetaxel-induced decrease in HIF-1α expression, whereas siPHD2 and siPHD3 were without effect (Fig. 3d), implicating PHD1 in docetaxel-induced suppression of HIF-1α expression. To confirm these data, we transfected MDA-MB-231 cells with siPHD1, siPHD2 or siPHD3, together with p5 × HRE-*luc* and pCMV-β-galactosidase. Cells were then treated with docetaxel for 16 h and exposed to 20% or 0.5% O_2_ for 4 h. Consistent with the results of immunoblot analyses, siPHD1 increased luciferase activity in docetaxel-treated cells (Fig. 3e). To confirm these data, we transfected MDA-MB-231 cells with siPHD1, siPHD2 or siPHD3, and the PHD-responsive promoter construct p*ODD-luc*. We then treated cells with docetaxel for 16 h and exposed them to 20% or 0.5% O_2_ for 4 h. We found that, under hypoxic conditions, luciferase activity was increased by siPHD1, but not by siPHD2 or siPHD3 (Fig. 3f), confirming the results of immunoblot analysis. Next, we investigated the post-translational modification responsible for the increase in the slower-migrating PHD1 band. First, phosphorylation was assessed by treating cells with λ**-**phosphatase. As shown in Fig. 3g, λ-phosphatase decreased the intensity of slower-migrating bands. To further demonstrate that docetaxel induces phosphorylation of PHD1 in cancer cells under hypoxia, we performed a mass spectrometry analysis. As shown in Fig. 3j, we detected phosphorylation of PHD1 at Ser-74 and Ser-162. In a previous report, inhibition of mitochondrial function was shown to allow PHDs to maintain their activity in low-oxygen environments^31^, supporting the possibility that docetaxel treatment reduces mitochondrial function, thereby decreasing oxygen consumption. Docetaxel treatment has also been shown to interfere with mitochondrial function and mitochondrial-dependent apoptosis^32^. To investigate whether mitochondrial function is impaired in docetaxel-treated cells, we exposed MDA-MB-231 cells to 0.5% O_2_ for 4 h in the absence or presence of 100 nM docetaxel, and monitored oxygen consumption and ATP generation. We found that docetaxel treatment reduced oxygen consumption up to 60% (Fig. 3h) and decreased ATP generation by 40% (Fig. 3i). These data indicate that docetaxel correlates with HIF-1α protein stability reduction in hypoxic cancer cells through phosphorylation (activation) of PHD1.

### Activation of JNK2 is required for PHD1-mediated HIF-1α degradation by docetaxel in cancer cells under hypoxia

**Figure 4:**
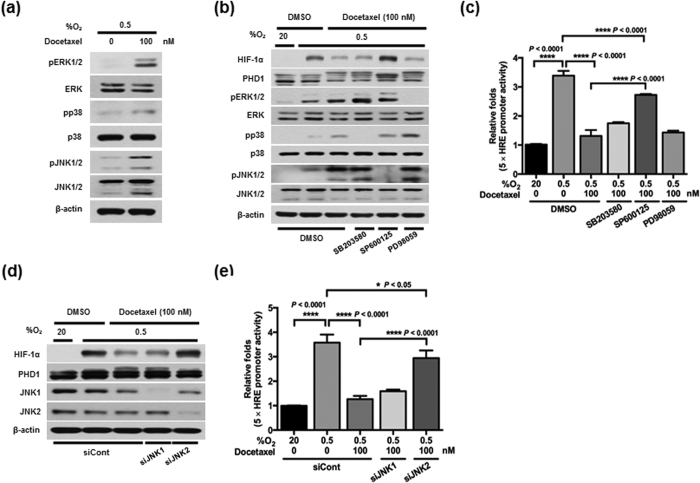
Activation of JNK2 is required for docetaxel-induced, PHD1-mediated HIF-1α degradation in cancer cells under hypoxia. (**a**) MDA-MB-231 cells were incubated with or without 100 nM docetaxel for 16 h, exposed to 0.5% O_2_ for 4 h, and harvested. Whole-cell lysates were analyzed by immunoblotting for the indicated proteins. **(b)** MDA-MB-231 cells were preincubated in the presence or absence of SB203580, SP600125 or PD98059 for 1 h, cultured with or without 100 nM docetaxel for 16 h, and exposed to 0.5% O_2_ for 4 h. Cell lysates were then analyzed by immunoblotting for the indicated proteins. **(c)** MDA-MB-231 cells co-transfected with p5 × HRE-*luc* and pCMV-β-galactosidase were cultured for 16 h, incubated with or without 100 nM docetaxel for 16 h in the presence or absence of SB203580, SP600125 or PD98059, and then exposed to 0.5% O_2_ for an additional 4 h. Luciferase activity was normalized to that of β-galactosidase. Data are presented as means ± SD (****P < 0.0001; ANOVA). **(d)** MDA-MB-231 cells were transfected with siRNA targeting JNK1 or JNK2, incubated with or without 100 nM docetaxel for 16 h, and exposed to 0.5% O_2_ for 4 h. Cells were harvested, and whole-cell lysates were analyzed by immunoblotting for the indicated proteins. **(e)** MDA-MB-231 cells co-transfected with siRNA targeting JNK1 or JNK2 together with p5 × HRE-*luc* and pCMV-β-galactosidase were cultured for 16 h, incubated with or without 100 nM docetaxel for 16 h, and exposed to 0.5% O_2_ for an additional 4 h. Luciferase activity was normalized to that of β-galactosidase. Data are presented as means ± SD (*P < 0.05, ****P < 0.0001; ANOVA).

Mitogen-activated protein kinase (MAPK) cascades play important roles in intracellular signaling in hypoxic cells^33^,^34^,^35^. Furthermore, docetaxel induces MAPKs in cancer cells^36^. To determine the potential involvement of MAPKs in docetaxel-induced PHD1 phosphorylation in cancer cells under hypoxia, we first used immunoblot analyses to examine MAPK protein and activation levels before and after docetaxel treatment. As shown in Fig. 4a, docetaxel increased the levels of phosphorylated ERK (extracellular signal-regulated kinase), p38, and JNK under hypoxic conditions. To determine which MAPK regulated the phosphorylation of PHD1, we pretreated cells with SP600125, PD98059, or SB203580, which inhibit JNK, MEK/ERK, and p38 MAPK, respectively. As shown in Fig. 4b, SP600125 effectively inhibited PHD1 phosphorylation and docetaxel-induced degradation of HIF-1α, whereas SB203580 and PD98059 had no effect. To confirm these data, we transfected MDA-MB-231 cells with p5 × HRE-*luc* and pCMV-β-galactosidase, treated them first with SP600125, PD98059, or SB203580 for 30 min and then with docetaxel for 16 h, and finally incubated them with 20% or 0.5% O_2_ for 4 h. As shown in Fig. 4c, SP600125 increased luciferase activity in docetaxel-treated cells, whereas SB203580 and PD98059 did not. To define the potential contribution of JNKs to HIF-1α regulation in docetaxel-treated hypoxic cells, we transfected MDA-MB-231 cells with siJNK1 or siJNK2, treated them with docetaxel for 16 h, and exposed them to 0.5% O_2_ for 4 h. As shown in Fig. 4d, siJNK2 prevented docetaxel-induced HIF-1α degradation by inhibiting phosphorylation of PHD1. To confirm these data, we transfected MDA-MB-231 cells with siJNK1 or siJNK2, together with p5 × HRE-*luc* and pCMV-β-galactosidase. We then treated the cells with docetaxel for 16 h, followed by exposure to 20% or 0.5% O_2_ for 4 h. Expression of siJNK2 increased HIF-1α–dependent transcriptional activity, confirming the results of immunoblot analyses (Fig. 4e). To complement these data, we investigated the effects of docetaxel on the expression of HIF-1α target genes (*Glut1, CA9, LDHA,* and *BNIP3*) involved in the metabolism of cancer cells and pro-apoptotic genes (*CASP3, CASP8,* and *CASP10*) in siJNK2-transfected MDA-MB-231 cells under hypoxic conditions. We found that JNK2 inhibition restored expression of HIF-1α target genes and suppressed expression of pro-apoptotic genes in docetaxel-treated cancer cells under hypoxic conditions (Fig. S1). Taken together, these results indicate that JNK2 is required for docetaxel-induced PHD1 activation and HIF-1α degradation.

### Docetaxel induces cancer cell death *in vitro* under hypoxic conditions

**Figure 5:**
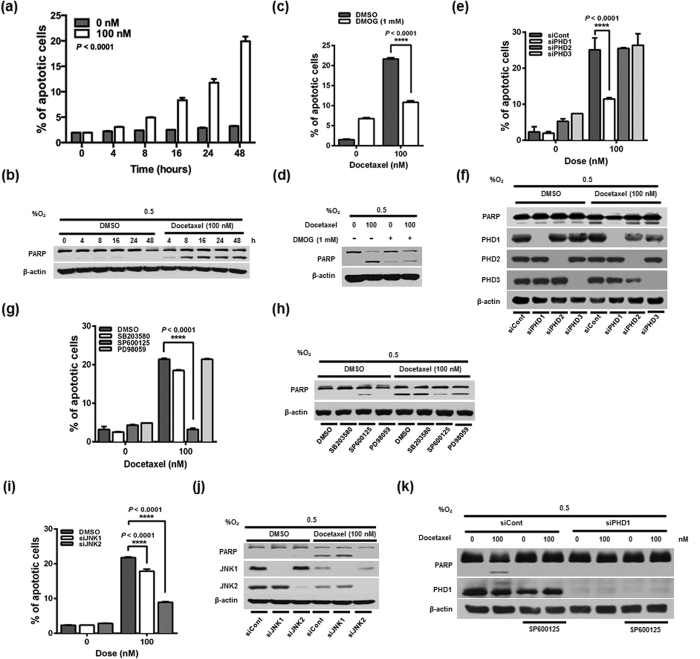
Docetaxel induces JNK2/PHD1-mediated cancer cell death under hypoxia. (**a**) Effect of docetaxel on the increase of apoptotic cell death in MDA-MB-231 cells under hypoxia. Apoptotic cell death was analyzed by flow cytometry using PI. Data are presented as means ± SD (****P < 0.0001; unpaired t-test). (**b**) Effect of docetaxel on PARP activation in MDA-MB-231 cells under hypoxia. (**c**) Effect of docetaxel on apoptotic cell death in MDA-MB-231 cells treated with or without DMOG (1 mM) under hypoxia. Apoptotic cell death was analyzed by flow cytometry using PI. Data are presented as means ± SD (****P < 0.0001; ANOVA). (**d**) Effect of docetaxel on PARP activation in MDAMB231 cells treated with or without DMOG under hypoxia. (**e**) Effect of docetaxel on PHD1-mediated cancer cell death in hypoxia. Apoptotic cell death was analyzed by flow cytometry using PI. Data are presented as means ± SD (****P < 0.0001; ANOVA). (**f**) Effect of docetaxel on PARP activation in MDA-MB-231 cells transfected with siRNA targeting PHD1, PHD2, or PHD3 under hypoxia. (**g**) Effect of docetaxel on JNK-mediated cancer cell death in hypoxia. Apoptotic cell death was analyzed by flow cytometry using PI. Data are presented as means ± SD (****P < 0.0001; ANOVA). (**h**) Effect of docetaxel on PARP activation in MDA-MB-231 cells treated with SB203580, SP600125 or PD98059. (**i**) MDA-MB-231 cells were transfected with siRNA targeting JNK1 or JNK2, incubated with or without 100 nM docetaxel for 16 h, and exposed to 0.5% O_2_ for 48 h. Apoptotic cell death was analyzed by flow cytometry using PI. Data are presented as means ± SD (****P < 0.0001; ANOVA) (**j**) MDA-MB-231 cells were transfected with siRNA targeting JNK1 or JNK2, incubated with or without 100 nM docetaxel for 16 h, and exposed to 0.5% O_2_ for 48 h. Whole-cell lysates were analyzed by immunoblotting for the indicated proteins. (**k**) MDA-MB-231 cells were transfected with siRNA targeting PHD1, treated with SP600125 for 1 h, incubated with or without 100 nM docetaxel for 16 h, and exposed to 0.5% O_2_ for 24 h. Whole-cell lysates were analyzed by immunoblotting for the indicated proteins.

To investigate the effects of docetaxel on cancer cell survival under hypoxic conditions, we treated MDA-MB-231 cells with 100 nM docetaxel for 16 h and exposed them to 0.5% O_2_ for 48 h. Cells were harvested at the indicated times and apoptotic cell death was analyzed using propidium iodide (PI) staining and flow cytometry. As shown in Fig. 5a, docetaxel treatment induced apoptosis in more than 20% of cells under hypoxic conditions, whereas <3% of control cells were apoptotic. To confirm that docetaxel induced apoptotic cell death, we evaluated cleaved poly(ADP-ribose) polymerase (PARP) by immunoblot analysis using an anti-PARP antibody. As shown in Fig. 5b, docetaxel induced a time-dependent increase in the levels of cleaved PARP under hypoxic conditions. Because docetaxel induces the JNK2/PHD1 signaling pathway and increases HIF-1α degradation (Figs 3 and 4), we investigated whether blocking the JNK2/PHD1 signaling pathway prevents docetaxel-mediated cancer cell death under hypoxic conditions through inhibition of HIF-1α degradation. We first investigated whether docetaxel-induced cancer cell death was caused by PHD1-dependent HIF-1α degradation. As shown in Fig. 5c,d, the PHD inhibitor, DMOG, reduced apoptotic cancer cell death (to ~10%) and inhibited PARP cleavage under hypoxic conditions. To define the potential contribution of PHDs to docetaxel-induced cancer cell death under hypoxic conditions, we transfected MDA-MB-231 cells with siPHD1, siPHD2 or siPHD3 for 24 h, treated these cells with docetaxel for 16 h, and exposed them to 0.5% O_2_ for 48 h. As shown in Fig. 5e,f, siPHD1 reduced apoptotic cancer cell death (to ~10%) and cleaved PARP, whereas siPHD2 and siPHD3 did not. Next, we treated MDA-MB-231 cells with SP600125, PD98059, or SB203580 to evaluate the contribution of JNKs to docetaxel-induced cancer cell death under hypoxic conditions. After pretreating with the inhibitors for 30 min, cells were treated with 100 nM docetaxel for 16 h and exposed to 0.5% O_2_ for 48 h. SP600125 reduced apoptotic cancer cell death (to ~10%) and cleaved PARP, whereas SB203580 and PD98059 did not (Fig. 5g,h). To further define the potential contribution of JNKs to docetaxel-induced cancer cell death under hypoxic conditions, we transfected MDA-MB-231 cells with siJNK1 or siJNK2, treated these cells with docetaxel for 16 h, and exposed them to 0.5% O_2_ for 48 h. As shown in Fig. 5i,j, siJNK2 reduced apoptotic cancer cell death (to ~10%) and levels of cleaved PARP; however, siJNK1 had no effect. Next, we investigated whether JNK2 and PHD1 act in the same pathway to increase docetaxel-induced cancer cell death under hypoxic condition. MDA-MB-231 cells were transfected with siJNK2 or negative control siRNA (siCont), treated with or without SP60015 for 1 h, then treated with or without 100 nM docetaxel for 16 h and exposed to 0.5% O2 for 24 h. As shown in Fig. 5k, SP60015 effectively inhibited docetaxel-induced increases in cleaved PARP, whereas siPHD1 did not inhibit docetaxel-induced PARP cleavage in the presence or absence of SP60015. Collectively, these findings indicate that docetaxel induces JNK2/PHD1 signaling under hypoxic conditions, leading to programmed cell death.

### Docetaxel-induced activation of the JNK2/PHD1 signaling pathway induces HIF-1α degradation and cancer cell death in a mouse xenograft model

**Figure 6:**
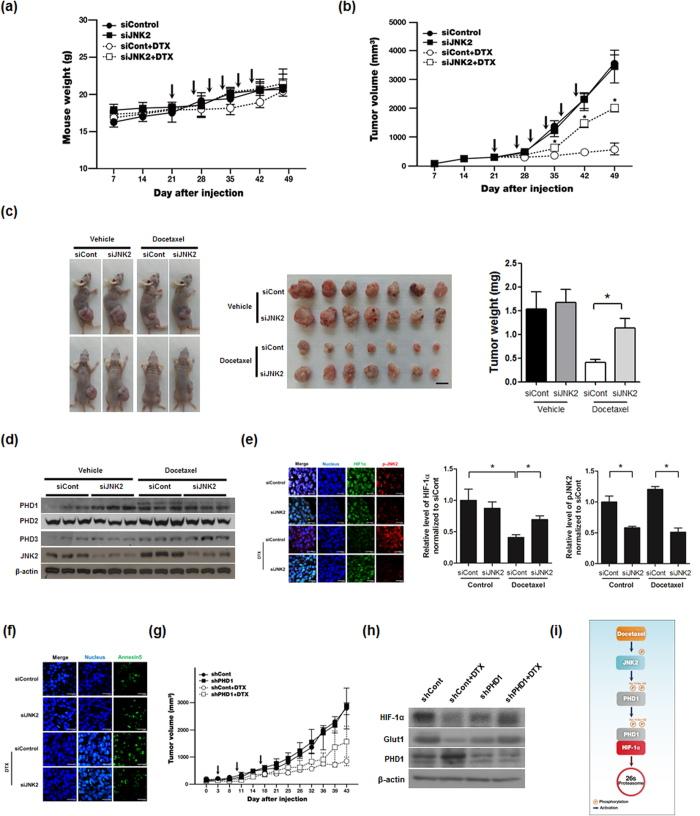
Docetaxel-induced activation of the JNK2/PHD1 signaling pathway induces HIF-1α degradation and cancer cell death in a mouse xenograft model. (**a**) Effect of docetaxel (DTX) on body weight during the course of the experiment. Arrows indicate treatment of DTX. (**b**) Effect of docetaxel-induced activation of the JNK2/PHD1 signaling pathway on tumor growth. Arrows indicate treatment of DTX. (**c**) *Left panel:* Representative images captured 7 week post-inoculation; *middle panel:* Images of individual MDA-MB-231 tumors at the time of sacrifice; *right panel:* Average tumor weight at sacrifice. (n = 7 animals/group; *P* < 0.05, unpaired t-test). (**d**) After mice were sacrificed, tumors were lysed and PHD1, PHD2, PHD3, JNK2, and β–actin were detected by immunoblot analysis. (**e**) *Left panel*: Immunofluorescence staining of HIF-1α and JNK2 in paraffin-embedded tissue specimens from mouse xenograft tumors. *Middle panel*: Signal intensities of HIF-1α were quantified using Image J. *Right panel*: Signal intensities of pJNK2 were quantified using Image J. (**f**) Apoptosis in tumor sections of the indicated experimental groups were determined using an *in situ* cell death detection kit. Data are presented as means ± SEM (n = 7; *P < 0.05, unpaired t-test). (**g**) MDA-MB-231/pshCont (shCont) and MDA-MB-231/pshPHD1 (shPHD1) cells (5 × 10^5^) were injected subcutaneously into the right flank of BALB/c nude mice. Once tumors had become established, they were treated with docetaxel (7.5 mg/kg/injection) weekly for 3 weeks. Tumor growth was assessed by calculating tumor volume using the following formula: Tumor volume = length × (width)^2^ × 0.5 (n = 3 animals/group; *P* < 0.05, unpaired t-test). Arrows indicate treatment of DTX. (**h**) After mice were sacrificed, tumors were lysed and PHD1, HIF-1α, GLUT1 and β–actin were detected by immunoblot analysis. (**i**) Schematic model showing how docetaxel induces cancer cell death under hypoxic conditions through JNK2/PHD1 signaling-mediated HIF-1α degradation.

Finally, to evaluate the effects of docetaxel on JNK2/PHD1 signaling-mediated HIF-1α degradation and cancer cell death *in vivo*, we used a nude mouse xenograft model, created by inoculation of MDA-MB-231 cells into the right flank of BALB/c nude mice. To inhibit the JNK2/PHD1 signaling pathway, we injected mice intratumorally with siJNK2 or siCont together with atelocollagen after tumors had reached a volume of ~100 mm^3^. As shown in Fig. 6a, mice treated with docetaxel showed no weight loss throughout the duration of the experiment. Following treatment of mice with docetaxel, siJNK2-transfected tumors displayed an increased growth rate, whereas siCont-transfected tumors grew much more slowly compared with untreated control tumors (Fig. 6b). After 7 weeks, mice were sacrificed and tumor weights were measured. Remarkably, tumor weights in docetaxel-treated mice bearing JNK2-knockdown MDA-MB-231 cells were 2.6-fold greater than those bearing siCont-transfected cells (Fig. 6c). The JNK2/PHD1 signaling-dependent HIF-1α degradation by docetaxel treatment in these tumors was further investigated (Fig. 6d,e). The PHD1 activation was suppressed and HIF-1α expression was increased in siJNK2-transfected tumors relative to those in siCont-transfected tumors after docetaxel treatment. In addition, siJNK2 effectively prevented docetaxel-induced cancer cell death (Fig. 6f). To confirm these findings, we studied the effect of docetaxel on the growth rate of shPHD1 (PHD1-knock-down) and shControl MDA-MB-231 xenografts in nu/nu mice. The growth rate of shPHD1 tumors was faster than that of shControl tumors (Fig. 6g). Consistent with these data, PHD1-knock-down effectively inhibited docetaxel-induced degradation of HIF-1α, thereby increasing expression of HIF-1α target gene, such as Glut1 (Fig. 6h). These data provide *in vivo* evidence that docetaxel-induced degradation of HIF-1α mediated by activation of the JNK2/PHD1 signaling pathway leads to programmed cell death under hypoxic conditions.

## Discussion

Here, we investigated the mechanisms underlying docetaxel-induced cancer cell death under hypoxic conditions. We found that docetaxel induces activation of JNK2, which mediates PHD1 phosphorylation. Activated PHD1, in turn, induces HIF-1α degradation, thereby causing cancer cell death in hypoxia.

Paclitaxel and docetaxel are known to increase the response of cancer cells to various chemotherapy drugs and radiation^1^,^4^,^5^,^6^,^7^. However, under hypoxic conditions, cancer cells are resistant to paclitaxel^37^, whereas they remain susceptible to docetaxel^4^. Consistent with these reports, expression of the pro-apoptotic genes, *CASP3, CASP8* and *CASP10*, was increased in docetaxel-treated cancer cells under both normoxic and hypoxic conditions, but not in paclitaxel-treated cancer cells under hypoxic conditions (Fig. S2). Previous studies have reported that hypoxia-induced autophagy promotes paclitaxel resistance in cancer cells^38^,^39^. We found that the autophagy markers, Beclin-1 and LC3-I/II, were increased in paclitaxel-treated cancer cells, but not in docetaxel-treated cancer cells (Fig. S3). Therefore, docetaxel is considered an effective agent for the treatment of paclitaxel-resistant cancer^40^.

It is well known that hypoxia-induced accumulation of HIF-1α allows cancer cells to survive oxygen deprivation, and induces angiogenesis, tumor growth, metastasis, chemoresistance, and radioresistance^9^,^10^,^11^,^12^,^13^,^14^. Thus, there is growing interest in using HIF-1α inhibitors as anticancer drugs^9^,^10^,^11^,^12^,^13^,^14^. In a previous report, docetaxel was shown to regulate hypoxia-induced HIF-1α protein expression; however, its mechanism of action remained unknown^9^. Consistent with this report, we found that docetaxel effectively inhibited HIF-1α accumulation (Fig. 1b,c). HIF-1α upregulates more than 70 target genes that regulate hypoxic adaptation and survival by binding to HREs in the promoter regions of these genes^8^,^10^,^11^,^26^. Here, we observed that docetaxel-induced inhibition of HIF-1α decreased HRE promoter activity by ~2-fold (Fig. 1f), thereby decreasing the expression of its target gene, *VEGF* (Fig. 1d,e). We further found that docetaxel-induced inhibition of HIF-1α increased hypoxic cancer cell death (Fig. 5a,b).

A previous study showed that docetaxel decreases HIF-1α accumulation, but does not alter mRNA levels^9^. After confirming that docetaxel regulates HIF-1α at the protein level (Figs 1b,c and 2b), we sought to determine how docetaxel regulates HIF-1α expression. Using CHX to block *de novo* HIF-1α synthesis, we found that docetaxel decreased HIF-1α protein stability (Fig. 2c), and further demonstrated that docetaxel increased HIF-1α degradation by inducing ubiquitination and subsequent proteasome-mediated degradation (Fig. 2d,e). Collectively, these findings indicate that docetaxel decreases HIF-1α protein stability in hypoxic cancer cells. In previous reports, HSP90 was shown to increase the stability of HIF-1α protein, whereas HSP40, HSP70, PHD1, PHD2, PHD3, and VHL decreased HIF-1α protein stability^13^,^14^. To delineate how docetaxel-induced HIF-1α degradation occurs in hypoxic cancer cells, we evaluated the expression of HIF-1α regulators. Interestingly, our immunoblot analyses showed that docetaxel increased the slower-migrating PHD1 band in cancer cells under hypoxic conditions (Fig. 3a). Using DMOG, a pharmacological inhibitor of PHD, and siRNAs targeting individual PHD isoforms, we investigated whether PHD1 participates in the regulation of HIF-1α in hypoxic cancer cells. We found that PHD1 inhibition partially restored HIF-1α accumulation and activation in docetaxel-treated cancer cells under hypoxic conditions (Fig. 3b–f) and reduced docetaxel-induced cell death (Fig. 5c–f). The PHD1-mediated degradation of HIF-1α requires oxygen, and a previous report showed that inhibition of mitochondrial function allows PHDs to maintain activity under hypoxic conditions^31^. Additionally, docetaxel may interfere with mitochondrial function^32^. Our data suggest that docetaxel impairs mitochondrial function, causing a decrease in oxygen consumption and ATP generation (Fig. 3h,i). Therefore, it appears that docetaxel-induced inhibition of oxygen consumption leads to moderate activation of PHD1, which in turn increases HIF-1α degradation (Fig. 3d–f,h,i). Our investigation of the post-translational modification responsible for the increase in the slower-migrating PHD1 band in docetaxel-treated cells revealed that the intensity of this band was decreased by treatment with λ-phosphatase (Fig. 3g), confirming the identity of this band as a phosphorylated form. Moreover, this decrease in the docetaxel-induced slower-migrating PHD1 band restored HIF-1α levels. In a previous report, Ortmann B. *et al.* demonstrated that cyclin-dependent kinase (CDK)-mediated phosphorylation of PHD1 at Ser-130 resulted in increased HIF-1α half-life by reducing PHD1-HIF-1α interaction^41^. In addition, another report suggested that protein kinase Cα increases phosphorylation of PHD1 at Ser-132, Ser-226 and Ser-234^42^. Interestingly, we found that docetaxel-induced JNK2 activation increased phosphorylation of PHD1 at Ser-74 and Ser-162 in hypoxic cancer cells (Fig. 3j). Collectively, our data demonstrate that docetaxel-induced PHD1 phosphorylation increases the degradation of HIF-1α (Fig. 3) and causes cancer cell death in hypoxic conditions (Fig. 5c–f). However, further research on phosphorylation of PHD1 at Ser-74 and Ser-162 is needed before any conclusion can be made regarding the importance of these phosphorylation sites in various cancer cells under hypoxic conditions.

Previous reports have indicated that MAPK cascades play an important role in intracellular signaling in hypoxic cells^33^,^34^,^35^. Additionally, microtubule-interfering agents including paclitaxel, docetaxel, vinblastine and nocodazole activate the JNK signaling pathway in cancer cells^36^. These microtubule-interfering agents activate both Ras and ASK1 signaling cascades, resulting in activation of JNK^36^.

Using specific pharmacological inhibitors and siRNAs to investigate the potential role of individual MAPKs in docetaxel-induced PHD1 activation in hypoxic cancer cells, we found that the JNK inhibitor SP600125 reduced PHD1 phosphorylation and restored HIF-1α accumulation (Fig. 4b,c). PHD1 inactivation increased HIF-1α transactivation (Fig. 4b,c) and effectively reduced docetaxel-induced cancer cell death under hypoxic conditions (Fig. 5g,h). Furthermore, siJNK2 effectively reduced PHD1 activation, thereby increasing HIF-1α accumulation and activation (Fig. 4d,e) and reducing docetaxel-induced cancer cell death under hypoxic conditions. Consistent with these results, docetaxel-treated JNK2-knockdown tumors grew much faster than siCont-injected tumors by virtue of inhibition of docetaxel-mediated PHD1 activation and degradation of HIF-1α. These results strongly suggest that docetaxel-induced activation of JNK2/PHD1 signaling increases HIF-1α degradation. In addition to HIF-1α, a recent study indicated that HIF-2α is also related to poor outcome in many malignancies^43^. Therefore, we investigated the effects of docetaxel on the expression of HIF-2α. Docetaxel-induced JNK2/PHD1 signaling effectively inhibited the expression of HIF-2α (Fig. S4).

Although the detailed mechanism by which JNK2 activates PHD1 remains unclear, our findings collectively demonstrate that docetaxel induces cell death in cancer cells under hypoxic conditions by activating the JNK2/PHD1 signaling pathway (Fig. 6i).

## Materials and Methods

### Cell lines and culture conditions

MDA-MB-231 human breast cancer cells, RKO human colorectal cancer cells, HeLa human cervical cancer cells, MCF7 human breast cancer cells, and SK-BR3 human breast cancer cells were cultured in Dulbecco’s Modified Eagle Medium (DMEM), RPMI-1640 medium or minimal essential medium (MEM), as recommended. Cells were incubated at 37 °C in a humidified 5% CO_2_ incubator unless otherwise noted. Hypoxic conditions were performed by incubation of cells in InvivO_2_ 500 hypoxia workstation (The Baker Company, Sanford, ME, USA). The oxygen concentrations were fixed with a gas mixture containing 5% carbon dioxide and balance nitrogen.

### Chemicals and antibodies

Docetaxel was purchased from Sigma-Aldrich (St. Louis, MO, USA). CHX and MG132 were purchased from Calbiochem (Darmstadt, Germany). DMOG was purchased from Santa Cruz Biotechnology (Dallas, TX, USA). Inhibitors specific for JNK (SP600125), ERK (PD98059) and p38 MAPK (SB203580) were purchased from Calbiochem. The indicated primary antibodies against the following proteins were used: HIF-1α (R&D Systems, Minneapolis, USA), HIF-1β (Cell Signaling Technology, Beverly, MA, USA), PHD1 (R&D Systems), PHD2 (Cell Signaling Technology), PHD3 (Novus Biologicals, Littleton, CO, USA), HSP90 (Enzo, Farmingdale, NY, USA), HSP70 (StressGen, Ann Arbor, MI, USA), HSP40 (StressGen), VHL (Cell Signaling Technology), β-actin (Sigma-Aldrich), VEGF (Santa Cruz Biotechnology, Santa Cruz, CA, USA), HA (Roche Applied Sciences, Indianapolis, IN, USA), ubiquitin (Invitrogen, Camarillo, CA, USA), pJNK (Cell Signaling Technology), JNK (Cell Signaling Technology), pERK (Cell Signaling Technology), ERK (Cell Signaling Technology), pp38 (Cell Signaling Technology), p38 (Cell Signaling Technology), and PARP (Cell Signaling Technology). Secondary antibodies used for immunoblotting included horseradish peroxidase (HRP)-conjugated anti-mouse (Cell Signaling Technology), anti-rabbit (Cell Signaling Technology), and anti-goat (Upstate, Darmstadt, Germany) antibodies. Mouse IgG was used as a negative control for immunoprecipitation (Santa Cruz Biotechnology).

### Construction of plasmids and stable cell lines

A plasmid encoding five tandem copies of an HRE from the *VEGF* regulatory region was constructed by ligating two oligonucleotides (5′-TCG AGC CAC AGT GCA TAC GTG GGC TCC AAC AGG TCC TCT TG-3′ and 5′-TCG ACA AGA GGA CCT GTT GGA GCC CAC GTA TGC ACT GTG GC-3′), as previously described (Guimbellot *et al.*, 2008), and subcloning this fragment into pGL3-Basic (Promega, Madison, WI, USA) upstream of the gene encoding firefly luciferase. The cloned plasmid was analyzed by restriction digestion and DNA sequencing (Bionics, Seoul, Republic of Korea). pHA-HIF-1α, pPHD1-EGFP and pODD-luc were purchased from Addgene (Cambridge, MA, USA). The pshPHD1 vector was constructed by cloning the appropriate double-stranded oligonucleotide (5′-TCT CGG ACA AGT ATC AGC TAG CAT CTT CCT GTC AAT GCT AGC TGA TAC TTG TCC CT-3′ and 5′-CTG CAG GGA CAA GTA TCA GCT AGC ATT GAC AGG AAG ATG CTA GCT GAT ACT TGT C-3′) into the pGeneClip vector (Promega). Stable MDA-MB-231 cell lines were generated by seeding cells at 5 × 10^4^ cells/well in 24-well plates and transfecting them with 50 μl of a mixture containing 1 μg of pshPHD1 together with the TurboFect *in vitro* transfection reagent (Fermentas, Hanover, MD, USA). The pGeneClip vector (Promega) was used as a control. Transfected cells were selected by culturing with 1 μg/ml puromycin (Sigma-Aldrich) for 1 week, and were maintained in RPMI-1640 containing 0.3 μg/ml puromycin during experiments.

### Dephosphorylation assay

Cells were incubated with or without 100 nM docetaxel and exposed to 20% or 0.5% O_2_. After 4 h, cells were harvested and lysed using lysis buffer (10 mM Tris-HCl pH 7.5, 150 mM NaCl, 1 mM EDTA, 0.1% NP-40, 1 mM PMSF) containing an inhibitor cocktail (Roche Applied Science). Lysed samples were incubated with 400 U of λ-phosphatase (New England Biolabs, Beverly, MA, USA) for 30 min at 30 °C. Proteins were resolved by sodium dodecyl sulfate-polyacrylamide gel electrophoresis (SDS-PAGE) and analyzed by immunoblotting.

### Immunoprecipitation of PHD1 and mass sprectrometry analysis

MDA-MB-231 cells were transfected with pPHD1-EGFP, treated with docetaxel (100 nM), exposed to 0.5% O_2_ for 16 h and harvested. Whole-cells were lysed and PHD1 was immunoprecipitated using anti-GFP antibody as described previously 41. Immunoprecipitation elutes were separated on SDS-PAGE gels and stained with commassie blue. The protein bands were excised and analyzed using Nano LC-LTQ-Orbitrap spectrometer (Korea Basic Science Institute, Chungbuk, Republic of Korea). Analysis of the collected data was performed by Sequest (v1.20).

### Transfection assays

Cells (5 × 10^4^), seeded into glass tubes with 2-cm^2^ flat walls (Bellco Glass; Catalog number #1908-16125) coated with 0.01% gelatin, were incubated overnight and co-transfected with 50 μl of a mixture containing 1 μg of p5 × HRE-*luc* or p*ODD-luc*, 0.1 μg of pCMV-β-galactosidase plasmid (transfection control; Stratagene, La Jolla, CA, USA), and TurboFect *in vitro* transfection reagent (Fermentas). After 16 h, cells were exposed to 20% or 0.5% O_2_ for 4 h, as described above. Luciferase activity was determined using a Luciferase assay system (Promega) and normalized to that of β-galactosidase, which was assessed using a β-galactosidase enzyme assay system (Promega) according to the manufacturer’s instructions. Three independent transfections were performed in each case. For immunoprecipitation and immunoblot analyses, cells were seeded into specially designed 25-cm^2^ glass flasks coated with 0.01% gelatin, incubated for 1d, and then transfected with expression vectors using the TurboFect *in vitro* transfection reagent, according to the manufacture’s protocol.

### RNA isolation and RT-PCR

Total RNA was extracted from MDA-MB-231 cells using the TRIzol reagent (Invitrogen) and treated with DNase I (New England Biolabs). cDNA was synthesized from total RNA (1 mg) using AccuPower RT PreMix (Bioneer, Daejeon, Republic of Korea), and the indicated target genes were PCR-amplified using the following primer pairs: *HIF-1*α, 5′-GTG TTA TCT GTC GCT TTG AGT-3′ (forward) and 5′-AAC TTC ACA ATC ATA ACT GGT-3′ (reverse); *VEGF*, 5′-AAC TTT CTG CTG TCT TGG-3′ (forward) and 5′-TTT GGT CTG CAT TCA CAT-3′ (reverse); *HIF-1β*, 5′-CAT CAG CTT CTG CAG GAC AG-3′ (forward) and 5′-GTA GCT GTT GCT CTG ATC TC-3′ (reverse); and 18S rRNA 5′-TCT CAG TTC CGT GGG TGG TG-3′ (forward) and 5′-TCG TTC GTT ATC GGA ATT AAC CAG-3′ (reverse). Primer pairs for *Glut1, LDHA, BNIP3, CA9, CASP3, CASP8* and *CASP10* were purchased from Bioneer. PCR products were analyzed by electrophoresis on 1.5% agarose/ethidium bromide gels.

### Immunoprecipitation and immunoblot analyses

Immunoprecipitation and immunoblot analyses were performed as we previously described^44^. For immunoprecipitation, cells were lysed using lysis buffer (10 mM Tris-HCl pH 7.5, 150 mM NaCl, 1 mM EDTA, 0.1% NP-40, 1 mM PMSF) containing an inhibitor cocktail (Roche Applied Science), sodium orthovanadate, and sodium fluoride. One milligram of total protein was immunoprecipitated using 1 μg of an appropriate antibody and collected with Protein G-magnetic beads (New England Biolabs) at 4 °C for 16 h. The immunoprecipitate was washed four times with cold lysis buffer, and bound proteins were resolved by SDS-PAGE and analyzed by immunoblotting. Immunoreactive proteins were detected by enhanced chemiluminescence (Pierce, Rockford, IL, USA).

### Measurement of O_2_ consumption

Cells (5 × 10^4^) were seeded into glass tubes with 2-cm^2^ flat walls coated with 0.01% gelatin and incubated overnight. Cells were incubated with or without 100 nM docetaxel and exposed to 0.5% O_2_ for 4 h. O_2_ consumption was determined using a Mito-ID O_2_ Extracellular Sensor Kit (Enzo), according to the manufacturer’s instructions, and normalized to protein concentration.

### ELISA analysis of secreted VEGF

Cells (5 × 10^4^) were seeded into glass tubes with 2-cm^2^ flat walls coated with 0.01% gelatin and incubated overnight. Cells were incubated with or without 100 nM docetaxel, exposed to 20% or 0.5% O_2_ for 1 h, as described above, and further incubated for 24 h. Supernatants were obtained and assayed using a VEGF (human) ELISA kit (Enzo) according to the manufacturer’s instructions.

### siRNA transfection

JNK1, JNK2, PHD1, PHD2 and PHD3 were knocked down by RNA interference using the following 19-bp (including a 2-deoxynucleotide overhang) siRNAs (Bioneer Corporation): JNK1, AAGCCCAGUAAUAUAGUAGUAdTdT; JNK2, CUGUAACUGUUGAGAUGUATTdTdT; PHD1, GACAAGUAUCAGCUAGCAUdTdT; PHD2, GAGUAGAGCAUAUAGAGAUdTdT; and PHD3, CGUGUAUCGUUC CCUCUdTdT. Stealth RNAi (Invitrogen) was used as a negative control (siCont). For transfection, cells were seeded in 25-cm^2^ flasks, grown to ~80% confluence, and transfected with siRNA duplexes using LipofectAMINE 2000 (Invitrogen) according to the manufacturer’s recommendations. After 48 h, the cells were processed as indicated for analysis.

### Ubiquitination assays

MDA-MB-231 cells were transfected with pHA-HIF-1α. Transfected cells were incubated with or without 100 nM docetaxel, exposed to 0.5% O_2_ for 4 h, and further incubated for 1 h in the presence or absence of 10 μg/ml MG132. After 1 h, cell samples were harvested and lysed with lysis buffer containing N-ethylmaleimide (Sigma-Aldrich). Supernatants were immunoprecipitated with an anti-HA antibody, and washed immunoprecipitates were resolved by SDS-PAGE and probed with an anti-ubiquitin antibody.

### ATP production

Cells (5 × 10^4^) were seeded into glass tubes with 2-cm^2^ flat walls coated with 0.01% gelatin and incubated overnight. Cells were incubated with or without 100 nM docetaxel and exposed to 0.5% O_2_ for 4 h. After removing the media, 100 μl of 0.3% trichloroacetic acid was added and cells were incubated for 30 minutes on ice. Protein precipitates were collected by centrifugation and discarded, and four volumes of 250 nmol/l Tris-acetate (pH 7.4) were added to the resulting supernatants. ATP content was determined using an ATP assay kit (Promega), according to the manufacturer’s instructions, and normalized to protein concentration.

### Quantification of cell death

Cells were collected by trypsinization, washed two times with phosphate-buffered saline (PBS), resuspended in 1 ml PBS containing 0.1% Triton X-100, 0.1 mM EDTA, 10 mg/ml DNase-free RNase A and 2 mg/ml PI, and incubated for 1 h in the dark at 37 °C. Apoptotic cells were detected by flow cytometry using a FACSCalibur system (Becton Dickinson, San Jose, CA, USA). Apoptosis was expressed as the percentage of cells in the sub-G1 population.

### *In vivo* mouse xenograft model

All procedures were carried out according to the Institutional Animal Care and Use Committee protocol approved for this study by Inha University (INHA 150605-363). Six-week-old, female nude mice (BALB/c-nu) were purchased from Orient Bio Laboratory Animal Inc. (Seoul, Korea) and maintained at 25 °C with a 12-h light/12-h dark cycle under specific pathogen-free conditions with ad libitum access to sterile water and food. For xenograft mouse models, MDA-MB-231 cells (1 × 10^6^) were mixed 1:1 with Matrigel (BD biosciences) in a total volume of 100 μl and injected subcutaneously into the right flank of mice (n = 7). After 1 week, mice were treated for 6 weeks (twice weekly) by direct, center-intratumoral injection of a 20-μmol/l dose of scrambled siRNA (siCont) or siJNK2, manufactured with atelocollagen^TM^ (Koken) to achieve effective delivery. Three weeks after tumor cell inoculation and confirmation of successful maturation of tumors (~100 mm^3^ in size), mice in the docetaxel group received docetaxel by intraperitoneal injection (2.5 mg/kg) twice weekly for 3 weeks, and tumor volume was measured by calipers every week. To evaluate the effect of docetaxel-induced activation of PHD1 in mouse model, MDA-MB-231/pshCont and MDA-MB-231/pshPHD1 cells (5 × 10^5^) were injected subcutaneously into the right flank of BALB/c nude mice. Once tumors had become established, the docetaxel treatement groups (shCont+DTX and shPHD1+DTX) received docetaxel by injection (7.5 mg/kg) weekly for 3 weeks. Six weeks after the first inoculation of tumor cells, all mice were sacrificed according to the animal experimental guidelines, and the developed tumors were excised and prepared using standard pathology procedures.

### Immunofluorescence staining

Paraffin-embedded whole-tissue sections were sectioned at 6 μm thickness and deparaffinized. Antigen retrieval was performed by heating in 0.1 mol/l sodium citrate (pH 6.0) using a microwave. Slides were then incubated overnight at 4 °C with a mixture of two primary antibodies, anti-HIF1α (Sigma) and anti-JNK2 (Cell Signaling), using antibody diluents (IHC World). Slides were rinsed with PBS and then incubated for 1 h with Alexa Fluor 594 or Alexa Fluor 488 (Life Technologies). Slides were mounted with ProLong Gold anti-fade reagent containing 4′,6-diamidino-2-phenylindole (DAPI; Thermo Fisher Scientific). Cell death was analyzed using the *in situ* cell death detection kit (Roche), following the manufacturer’s instructions. Confocal images were obtained using a Nikon D-Eclipse C1 confocal system and processed with Nikon EZ-C1 3.90 and Adobe Photoshop software.

### Statistical analysis

All immunoblots are representative of at least three separate experiments. All grouped data are presented as mean ± SD. Differences between groups were analyzed by analysis of variance (ANOVA) or Student’s t test, as appropriate, using GraphPad Prism software. All experiments were repeated in at least duplicate with triplicate technical replicates.

## Additional Information

**How to cite this article**: Oh, E.-T. *et al.* Docetaxel induced-JNK2/PHD1 signaling pathway increases degradation of HIF-1α and causes cancer cell death under hypoxia. *Sci. Rep.* **6**, 27382; doi: 10.1038/srep27382 (2016).

## Supplementary Material

Supplementary Information
