# Supplementary material for: Docetaxel induced-JNK2/PHD1 signaling pathway increases degradation of HIF-1α and causes cancer cell death under hypoxia
Source: Sci Rep. 2016 Jun 6;6:27382. doi: 10.1038/srep27382 (PMC4893693; doi:10.1038/srep27382)
Supplement: Supplementary Information [file srep27382-s1.pdf]

Docetaxel induced-JNK2/PHD1 signaling pathway increases degradation of HIF-1α and causes cancer cell death under hypoxia

Eun-Taex Oh[1,2], Chan Woo Kim[3], Soo Jung Kim[1], Jae-Seon Lee[2,4], Soon-Sun Hong[1,2,*] & Heon Joo Park[2,3,*]

[1]Department of Biomedical Sciences, College of Medicine, Inha University, Incheon 22212, Republic of Korea, [2]Hypoxia-related Disease Research Center, College of Medicine, Inha University, Incheon 22212, Republic of Korea, [3]Department of Microbiology, College of Medicine, Inha University, Incheon 22212, Republic of Korea, [4]Department of Molecular Medicine, College of Medicine, Inha University, Incheon 22212, Republic of Korea

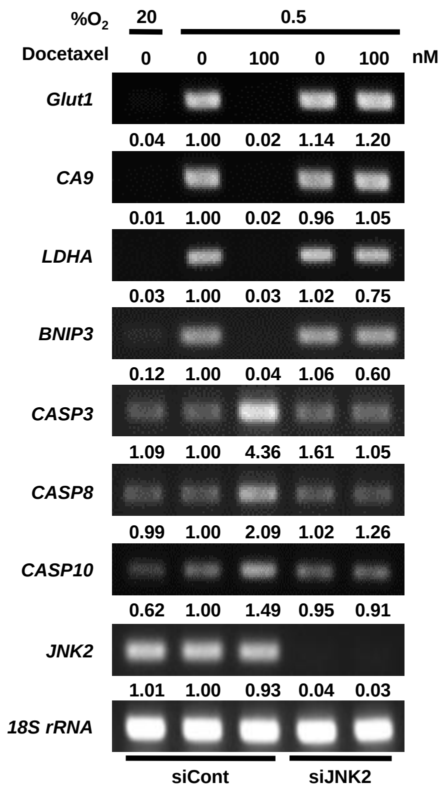

Figure S1. Effect of JNK2 on docetaxel-induced suppression of the expression of HIF-1α target genes involved in the metabolism of cancer cells under hypoxia. MDA-MB-231 cells were transfected with siRNA targeting JNK2, incubated with or without 100 nM docetaxel for 16 h, exposed to 0.5% O_2 for 24 h, and then harvested. *Glut1*, *CA9*, *LDHA*, *BNIP3*, *CASP3*, *CASP8*, *CASP10* and *JNK2* mRNAs and *18S rRNA* were amplified by RT-PCR. Band intensities of RT-PCR products were quantified using Image J (NIH).

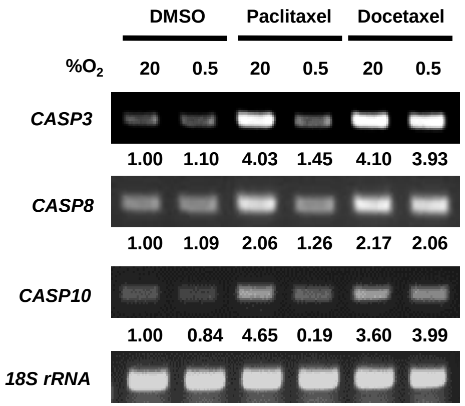

Figure S2. Effect of docetaxel on the expression of pro-apoptotic genes. MDA-MB-231 cells were treated with or without paclitaxel (50 μM) or docetaxel (100 nM), incubated under normoxic or hypoxic conditions, and then harvested. *CASP3, CASP8* and *CASP10* mRNAs and *18S rRNA* were amplified by RT-PCR. Band intensities of RT-PCR products were quantified using Image J.

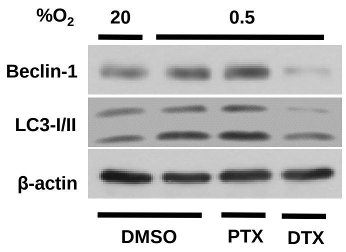

Figure S3. Effect of docetaxel on the expression of autophagy markers in cancer cells under hypoxia. MDA-MB-231 cells were treated with or without paclitaxel (5 μM) or docetaxel (100 nM), exposed to 0.5% O_2 for 16 h and harvested at the indicated times. Whole-cell lysates were analyzed by immunoblotting for the indicated proteins.

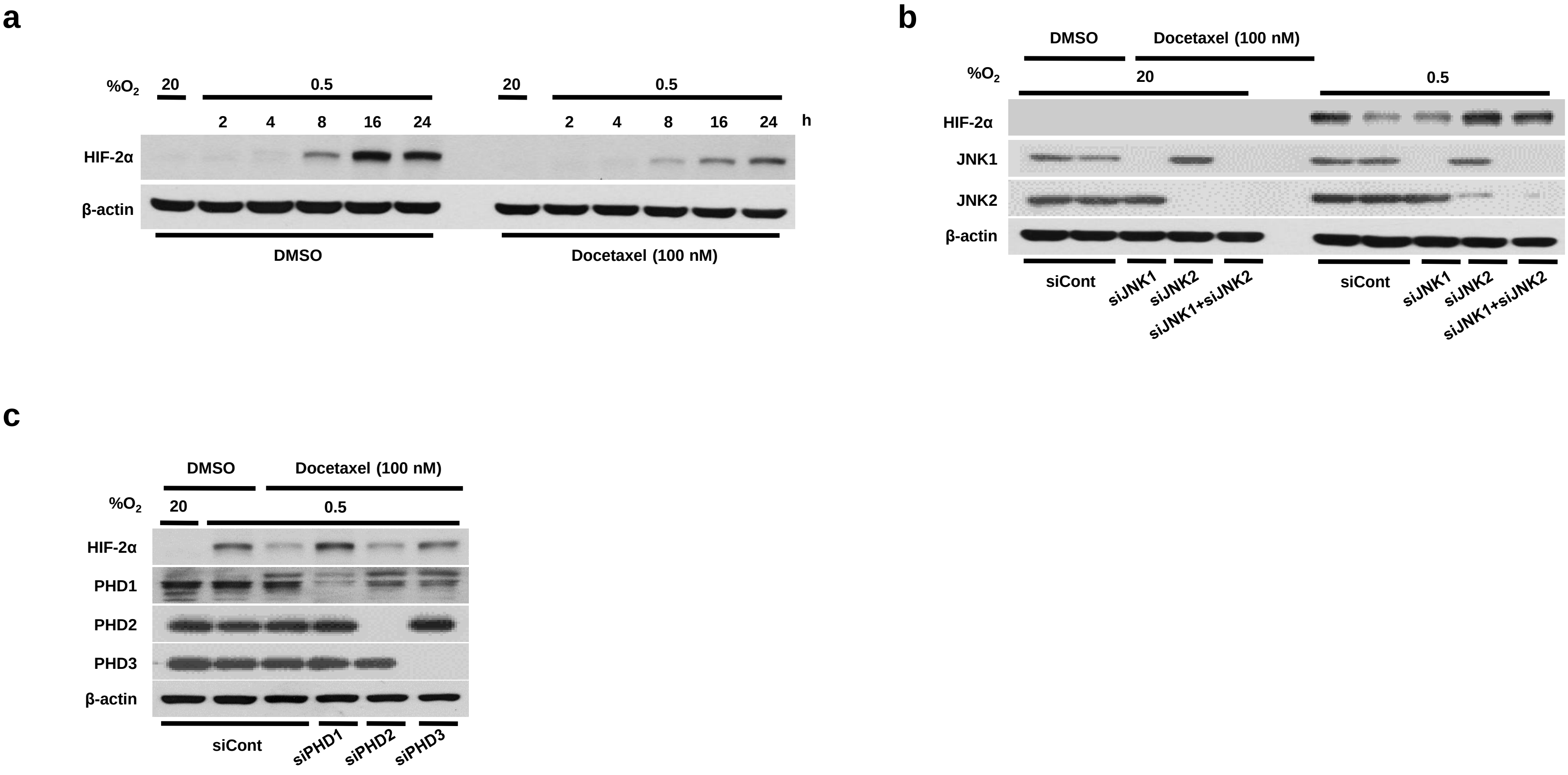

Figure S4. Effect of docetaxel on the expression of HIF-2α. (a) MDA-MB-231 cells were treated with or without 100 nM docetaxel for 16 h, exposed to 0.5% O_2 for 24 h, and harvested at the indicated times. Whole-cell lysates were analyzed by immunoblotting for the indicated proteins. **(b)** MDA-MB-231 cells were transfected with siRNA targeting JNK1 or JNK2, incubated with or without 100 nM docetaxel for 16 h, and exposed to 0.5% O_2 for 8 h. Cells were harvested, and whole-cell lysates were analyzed by immunoblotting for the indicated proteins. **(c)** MDA-MB-231 cells were transfected with siRNA targeting PHD1, PHD2 or PHD3, incubated with or without 100 nM docetaxel for 16 h, and exposed to 0.5% O_2 for 8 h. Cells were harvested, and whole-cell lysates were analyzed by immunoblotting for the indicated proteins.
